# Molecular Typing of *Enterobacteriaceae* from Pig Holdings in North-Western Germany Reveals Extended- Spectrum and AmpC β-Lactamases Producing but no Carbapenem Resistant Ones

**DOI:** 10.1371/journal.pone.0134533

**Published:** 2015-07-30

**Authors:** Silvia García-Cobos, Robin Köck, Alexander Mellmann, Julia Frenzel, Alexander W. Friedrich, John W. A. Rossen

**Affiliations:** 1 Department of Medical Microbiology, University of Groningen, University Medical Center Groningen, Groningen, The Netherlands; 2 Institute of Medical Microbiology, University Hospital Münster, Münster, Germany; 3 Institute of Hygiene, University Hospital Münster, Münster, Germany; Amphia Ziekenhuis, NETHERLANDS

## Abstract

The increase of extended- spectrum β-lactamase-producing *Enterobacteriaceae* (ESBL-E) in humans and in food-producing animals is of public health concern. The latter could contribute to spreading of these bacteria or their resistance genes to humans. Several studies have reported the isolation of third generation cephalosporin resistant bacteria in livestock animals. However, the number of samples and the methodology used differ considerably between studies limiting comparability and prevalence assessment. In the present study, a total of 564 manure and dust samples were collected from 47 pig farms in Northern Germany and analysed to determine the prevalence of ESBL-E. Molecular typing and characterization of resistance genes was performed for all ESBL-E isolates. ESBL-E isolates were found in 55.3% of the farms. ESBL-*Escherichia coli* was found in 18.8% of the samples, ESBL-*Klebsiella pneumoniae* in 0.35%. The most prevalent ESBL genes among *E*. *coli* were CTX-M-1 like (68.9%), CTX-M-15 like (16%) and CTX-M-9 group (14.2%). In 20% of the latter two, also the OXA-1 like gene was found resulting in a combination of genes typical for isolates from humans. Genetic relation was found between isolates not only from the same, but also from different farms, with multilocus sequence type (ST) 10 being predominant among the *E*. *coli* isolates. In conclusion, we showed possible spread of ESBL-E between farms and the presence of resistance genes and STs previously shown to be associated with human isolates. Follow-up studies are required to monitor the extent and pathways of ESBL-E transmission between farms, animals and humans.

## Introduction

In the last decade the emergence of plasmid-encoded AmpC- β-lactamase- and extended-spectrum β-lactamase (ESBL)-producing *Enterobacteriaceae* among livestock animals has raised concern that food-producing animals may contribute to zoonotic spread of these antibiotic-resistant bacteria to humans [1–4]. Indeed, persons in the general population are increasingly colonized with ESBL-producing *Enterobacteriaceae* (ESBL-E). Recent studies in Germany demonstrated that ESBL-E carriage affects 4–6% of all humans [5–7].

Zoonotic dissemination of resistant bacteria could either result from direct transmission between livestock animals and farmers [6, 8, 9], or via the introduction of ESBL-E in the food chain [10]. Several studies have proven that European retail meat and vegetables are contaminated with ESBL-E [11–14]. Furthermore, some studies indicated similarities between *Escherichia coli* strains and associated resistance genes found in meat and humans [12, 15]. In addition, spread of ESBL-encoding plasmids between bacteria from human sources and food-producing animals have been reported [16–21].

The proportion of the total burden of human colonization or infection with ESBL-E linked to the livestock reservoir is still controversial, because source attribution results are difficult to interpret [5, 22]. This is mainly due to the fact that the most accurate method for tracing epidemiological pathways of ESBL-E dissemination is unclear. Differently from multidrug-resistant microorganisms following a clonal distribution pattern, such as methicillin-resistant *Staphylococcus aureus*, it seems inadequate to exclude a possible link between two ESBL-E isolates if they belong to two different clonal lineages, as antibiotic-resistance genes, located on mobile genetic elements, can be transferred between clones. Furthermore, it is unknown to what extent transfer of different resistance-associated mobile genetic elements occurs at random, or if specific elements are more prone to be acquired by enterobacteria colonizing the human intestine or causing human infection.

As a consequence, molecular characterization of ESBL-E isolates from food-producing animals has become a major research topic aiming to understand zoonotic transmission as well as transmission of ESBL-E among food-producing animals. International studies among livestock animals including poultry, cattle and pigs indicate that most ESBL-E are *E*. *coli* associated to multilocus sequence typing (MLST) clonal complexes (CC) 648, 23, 10, 405, 131, 69, and 73, and that CTX-M-1 is the most frequently detected ESBL gene [2, 3]. Other ESBL or AmpC β-lactamases frequently found in livestock animals are CMY-2, TEM-52, and SHV-12 [2, 23].

Germany is one of the major European producers of pig meat. On German pig farms, occurrence of cefotaxime resistant *E*. *coli* [24] or the occurrence of ESBL/AmpC- producing *E*. *coli* has been previously reported ranging in prevalence from 43.8% in fattening pig holdings to 56.3%, if individual fattening pigs are considered [25]. Currently published investigations on molecular typing results for ESBL-E isolates from German pig farms indicate that in *E*. *coli* isolates CTX-M-1 (66.7%) is the most frequent ESBL-gene followed by CTX-M-9 (3.5%) and CTX-M-15 (3.5%) [26]. However, as recent studies have focussed on assessing resistance genes, phylogenetic groups or PFGE profiles of ESBL-associated *E*. *coli* isolates [22, 24–26], there is limited knowledge on associated clonal lineages as determined by MLST.

Here we aim to get new insights into the prevalence of ESBL/AmpC-producing bacteria in pig farms in Germany. The following specific objectives were addressed: i) to study the β-lactamase genes present in cefotaxime resistant *Enterobacteriaceae* isolated from pig holdings in North-western Germany; ii) to compare possible outbreak-associated strains between farms using repetitive-sequence-based PCR (rep-PCR); and iii.) to determine the MLST in randomly selected isolates to reveal the clonal background of the bacterial collection in a global context [27].

## Materials and Methods

### Farms and sample collection

A total of 47 pig farms participated to investigate the prevalence of ESBL-E in swine holdings in the German part of the Euregio (comprising the German federal states of Lower Saxony and North Rhine-Westphalia (NRW). Between February 2013 and September 2013, six dust samples (collected using Polywipe sponge swabs, Westerau, Germany) and six manure samples (collected using sterile cotton swabs) were obtained from different locations on each pig farm. Sampling was performed by veterinarians from the animal health services of the Federal Chambers for Agriculture of the states of NRW and Lower Saxony. Farms were not actively selected, but samples were derived during routine visits of the animal health services on the farms. Of each location, one dust sample (n = 282) and one manure swab (n = 282) were enriched in non-selective broth (Caso broth, Heipha, Eppelheim, Germany) and incubated for 24h at 35 +/- 1°C. Ten μl of the sample was streaked onto a chromogenic medium for the detection of ESBL-producing organisms (bioMérieux, Marcy l’Etoile, France).

### Species identification and susceptibility testing

Presumptive ESBL colonies were subcultured on blood agar. Species confirmation was done by MALDI-TOF (Bruker Daltonik GmbH, Bremen). Susceptibility testing for ESBL-E was done by VITEK 2 automated systems according to EUCAST clinical breakpoints [28].

### Resistance genotyping

ESBL-E positive samples were selected for DNA extraction using the UltraClean Microbial DNA Isolation Kit (MoBio, Laboratories, Inc.) and further characterized for the presence of ESBL/AmpC genes using a DNA-array (Check-MDR CT103, Check-points, Wageningen, The Netherlands) that includes specific DNA markers to identify the presence of the ESBL genes TEM, SHV and CTX- M (subgroups belonging to the CTX-M-1 group: CTX-M- 1 like, CTX-M-15 like, CTX-M-3 like and CTX-M-32 like); and discriminates between ESBL and non-ESBL TEM and SHV variants by detecting Single Nucleotide Polymorphisms (SNPs) corresponding to amino acid positions 104, 164 and 238 in TEM, and 238 and 240 in SHV. It also detects pAmpC (CMY-2-like, DHA, FOX, ACC-1, ACT/MIR and CMY-1-like/MOX) and carbapenemases (KPC, OXA-48, VIM, IMP and NDM) genes [29]. Besides, the positive and negative controls included in the kit, a clinical ESBL- producing *E*. *coli* isolate was included as a positive control.

Additionally, samples positive for CTX-M-15 like or CTX-M-9 group were assessed by PCR for the presence of *bla*_OXA-1_ gene, which is not detected by the DNA-array. This gene has been previously described to be associated with the presence of CTX-M-15 in humans resulting in resistance to inhibitors [30]. The PCR amplified part of the gene (between nucleotide positions bp 54 and 321) using the following oligonucleotide primer pairs: forward (5’ TAT CTA CAG CAG CGC CAG TG 3’) [31] and reverse primer (5’ GCT GTT CCA GAT CTC CAT TC 3’), designed in this study. PCR conditions were as follows: denaturation at 94°C for 5 min followed by 35 cycles of denaturation at 94°C for 1 min, annealing of primers at 59°C for 1 min, and primer extension at 72°C for 1 min, followed by a final primer extension step at 72°C for 5 min. Reactions were performed in a 25-μl final volume in duplicate, with the ReadyMix Taq PCR Reaction Mix method (Sigma-Aldrich Co. LLC), containing 1 and 5 μl of DNA template, 10 mM Tris-HCl (pH 9), 50 mM KCl, 1.5 mM MgCl_2_, 1 μM concentrations of each primer, 200 μM concentrations of each deoxynucleoside triphosphate, and 2.5 U of *Taq* polymerase. PCR products were visualized by 1% agarose gel electrophoresis and SYBR Green staining.

### Rep-PCR

Clonal relatedness among *E*. *coli* isolates was determined by the Diversilab (DL) bacterial typing system according to the manufacturer’s instructions (bioMérieux, Marcy l’Etoile, France). Analysis was performed using Pearson correlation in the dedicated DL software of the manufacturer (version 3.4). Isolates with a similarity <90% were considered different and isolates with a similarity >95% were considered indistinguishable. All isolates with a similarity between 90% and 95% were judged visually.

### *E. coli* MLST

MLST was carried out on one isolate of every cluster obtained by DL as described previously [32]. Although the exact ST cannot be inferred for all isolates belonging to the same DL cluster, it was used as a first screening to determine the presence of clinical important STs [33]. Allelic profiles and STs were assigned in accordance to the *E*. *coli* MLST website [34].

### Statistical analysis

Differences in the prevalence of ESBL-E between regions were assessed by the Fisher exact test. Statistical analyses were performed using GraphPad Prism version 6 (GraphPad Prism Software, Inc.).

### Ethics statement

The study was performed on private land. The owner of the farm provided informed consent. No specific permissions were required for these locations (except permission of the owner). Studies did not involve endangered or protected species. Only environmental samples were included in the study.

## Results

The prevalence of ESBL-E on farm level was 55.3% (26/47 positive farms) and no differences were found between the two federal states involved (Fisher’s exact test, *p* = 0.12). Of the total manure (n = 282) and dust (n = 282) samples tested, 27.3% (n = 77) and 10.3% (n = 29) were positive for ESBL-*E*. *coli*, respectively. In addition, 0.7% (n = 2) of dust samples were positive for ESBL-*Klebsiella pneumoniae*.

### Antibiotic susceptibility

Results on antibiotic susceptibility data for *E*. *coli* isolates are shown in Table 1. Regarding β-lactam inhibitors, 84% and 4.7% of the *E*. *coli* isolates, were ampicillin/sulbactam and piperacillin/tazobactam resistant, respectively. The two *K*. *pneumoniae* isolates found were resistant to ampicillin, ampicillin/sulbactam, cefuroxime, cefotaxime and trimethoprim/sulfamethoxazole, and intermediate to piperacillin/tazobactam and ceftazidime (data not shown). All *E*. *coli* and *K*. *pneumoniae* isolates were susceptible to carbapenems.

**Table 1:** Antibiotic susceptibility data of 106 *E*. *coli* isolates from 47 German pig farms.

	No. of isolates (% of 106 *E*. *coli* isolates)		
Antibiotic	Susceptible	Intermediate	Resistant
Ampicillin	0 (0)	0 (0)	106 (100)
Ampicillin-sulbactam	7 (6.6)	0 (0)	89 (84)
Piperacillin-tazobactam	2 (1.9)	99 (93.4)	5 (4.7)
Cefuroxime	0 (0)	0 (0)	106 (100)
Cefotaxime	0 (0)	0 (0)	106 (100)
Ceftazidime	69 (65.1)	22 (20.8)	15 (14.2)
Ertapenem	106 (100)	0 (0)	0 (0)
Imipenem	106 (100)	0 (0)	0 (0)
Meropenem	106 (100)	0 (0)	0 (0)
Gentamycin	94 (88.7)	0 (0)	12 (11.3)
Ciprofloxacin	78 (73.6)	0 (0)	28 (26.4)
Moxifloxacin	76 (71.7)	2 (1.9)	28 (26.4)
Tetracycline	31 (29.2)	0 (0)	75 (70.8)
Trimethoprim-sulfamethoxazole	38 (35.8)	0 (0)	68 (64.2)

### β-lactamase genes

The β-lactamase genes found in *E*. *coli* isolates are shown in Table 2.The most prevalent ESBL gene was a CTX-M-1-like gene, alone (43.4%, n = 46) or in combination with TEM_non-ESBL_ (25.5%, n = 27). In addition, 6 (5.7%) *E*. *coli* isolates had an OXA-1 like gene. Two *E*. *coli* isolates presented a plasmid AmpC CMY-II together with TEM_non-ESBL_. One *E*. *coli* isolate only harboured TEM_non-ESBL_ conferring ampicillin-resistance. For this isolate the mechanism causing the observed resistance against cefotaxime remained unclear. The two *K*. *pneumoniae* isolates harboured a CTX-M-1-like and SHV_non-ESBL_ gene.

**Table 2:** Distribution and combinations of β-lactamase genes in 106 *E*. *coli* identified from 47 German pig farms.

β-lactamase gene	No. of isolates (% of 106 *E*. *coli* isolates)
CTX-M-1 like	46 (43.4)
CTX-M-1 like + TEM_non-ESBL_	27 (25.5)
CTX-M-15 like	7 (6.6)
CTX-M-15 like + TEM_non-ESBL_	4 (3.8)
CTX-M-15 like + OXA-1 like	1 (1)
CTX-M-15 like + TEM_non-ESBL_+OXA-1 like	3 (2.8)
CTX-M-9 group	6 (5.7)
CTX-M-9 group + TEM_non-ESBL_	7 (6.6)
CTX-M-9 group + TEM_non-ESBL_ +OXA-1 like	2 (1.9)
CMY-II + TEM_non-ESBL_	2 (1.9)
TEM_non-ESBL_	1 (1)

### Rep-PCR and MLST analysis

**Fig 1:**
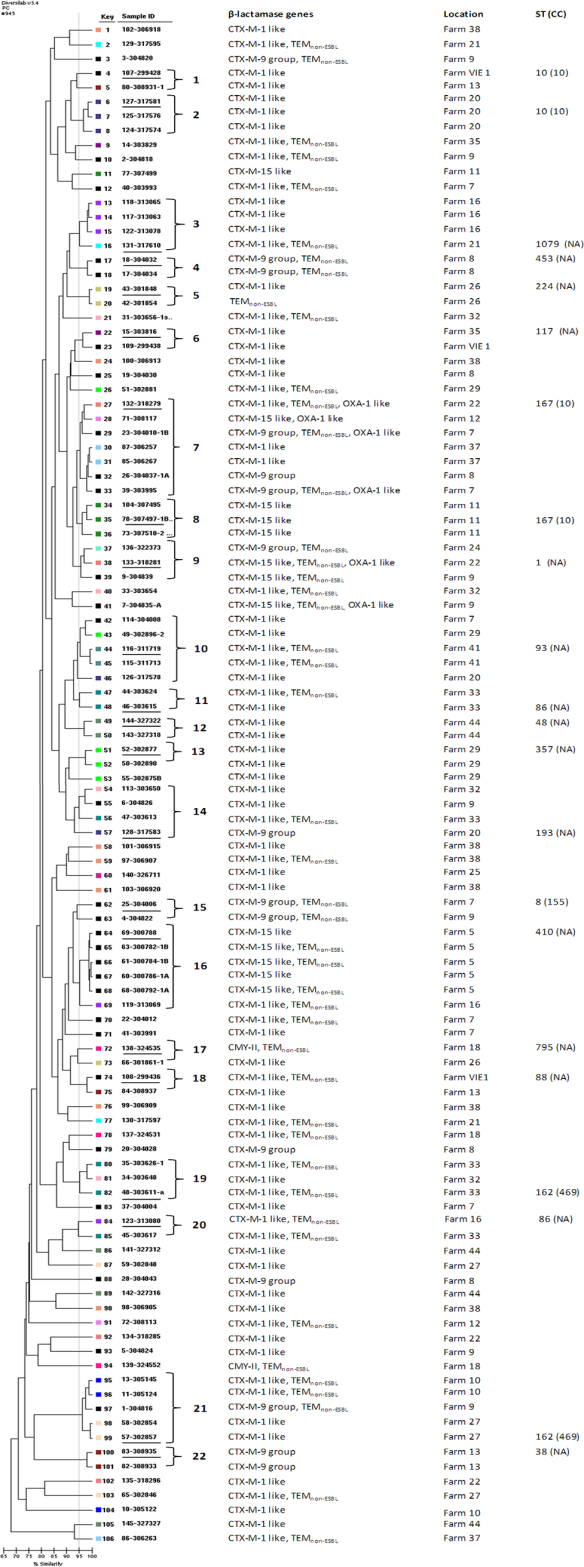
Dendrogram displaying the genetic relatedness of 106 *E*. *coli* isolates from pig holdings in Germany. The dendrogram was constructed by Diversilab (DL) software. Type designations for DL clusters are given as numbers on the right. Singletons are left without designations. MLST was performed in underlined isolates and coloured boxes indicate farm numbers. The vertical grey line indicates 95% of similarity. NA, not assigned to a CC.

The genetic relationship among *E*. *coli* isolates is shown in the dendrogram of the Rep-PCR results (Fig 1). A total of 22 clusters formed by more than one isolate with an intracluster pairwise similarity ≥ 95%, were observed. Eight clusters were formed by isolates from the same farms, seven of them contained isolates with the same CTX-M β-lactamase gene. Fourteen clusters were formed by isolates from different farms, eight of them contained isolates with the same CTX-M β-lactamase gene (Fig 1). One isolate of every cluster was studied by MLST, most clusters showed different STs, except cluster 1 and 2 (ST10, CC10), cluster 7 and 8 (ST167, CC10), and cluster 19 and 21 (ST162, CC469) (Fig 1).

## Discussion

An increasing number of reports on the occurrence of multiresistant bacteria in livestock animals and food items at retail are published. However, controversial information is available on the prevalence and genetic relatedness with human isolates. This is a large study into ESBL prevalence in pig holdings in Germany also including data on resistance genes and clonality. The high percentage of farms (55.3%) observed on ESBL-producing bacteria, which may even be underestimated due to the use of environmental samples and non-selective enrichment [35], indicates the importance of implementing measures to monitor and control the spread of these bacteria within the veterinary setting.

Most ESBL-positive samples contained an *E*. *coli* (98.1%). In addition, also ESBL-producing *K*. *pneumoniae* isolates (1.9%) were found that have so far not been reported in studies on livestock animals in Europe. Only one report from Korea describes five non-ESBL *K*. *pneumoniae* isolates in pigs [2, 36]. Importantly, no carbapenem resistance was found in this collection of isolates in contrast to what has been reported in two fattening pig farms positive for VIM-1 producing *Salmonella* in another German study [37].

The predominant ESBL gene family in this *E*. *coli* collection was CTX-M (97.2%), mostly CTX-M-1 like followed by CTX-M -15 like and CTX-M-9 group. These findings are in accordance with other studies in German pigs, although in some cases no data on specific CTX-M genes was given or only a low number of isolates was tested [25, 26, 38, 39]. Also, studies in livestock animals, including pigs, in other European countries reported CTX-M as the most common found ESBL genes [40–42]. Although CTX-M-15 and CTX-M-14 seem to be the major types in humans [2], CTX-M-1 has also been described as a prevalent ESBL gene in human source isolates [43].

Of all isolates 1.9% harboured CMY-II, a plasmid-AmpC most predominant among poultry but also described in other hosts [2]. It has been suggested that the detection of CMY-II is related with the presence of possible neighboring cattle or poultry farms in the proximity [26]. Unfortunately, no such data are available for the present study.

Remarkably, we detected one isolate harbouring only TEM_non-ESBL_ and no ESBL genes, which still showed cefotaxime cephalosporin resistance. The observed resistance pattern may be due to the hyperproduction of a chromosomal AmpC present in *E*. *coli* [44] or to a yet unidentified mechanism.

In this study, we confirmed investigations on the rare occurrence of OXA-1 genes in German pigs. The gene has been shown to be present in *E*. *coli* isolates from poultry meat, companion animals, cattle and waterbirds and also in *Samonella* spp. (mainly retail meat samples) [45–51]. In the present study, the OXA-1 like gene was studied in CTX-M-15 or CTX-M-9 producing isolates that are more linked to the human population. Indeed, CTX-M-15 and OXA-1 like have been described to be associated with the same plasmid, pC15 [52], suggesting that successful spread of some antibiotic resistance genes is due to transmission of epidemic plasmids.

Moreover, the presence of highly-related plasmids carrying ESBL and AmpC genes among *E*. *coli* isolates from different reservoirs has recently been described using whole genome sequencing. Transmission has been proven between human- and pig- but not between human- and poultry-associated isolates due to the considerable heterogeneity of the strains and despite the fact that in both cases isolates from different sources were considered to be identical based on MLST, plasmid typing or antibiotic resistance gene sequencing [12, 15,16]. Other studies support this plasmid-mediated spread of resistance genes between healthy humans and animals [53]. These studies give more insight into the possible mechanisms of ESBL dissemination and indicate that transmission of strains between different origins may be less frequent, but that the easy transfer of mobile genetic elements between bacteria of the same or even different genera is an important factor in the spread of antibiotic resistance genes. More studies including strains from different sources, animal, humans and food are needed to elucidate this pathway.

The dendrogram obtained by rep-PCR showed several clusters comprising isolates harbouring the same ESBL genes and from the same farm. This is not surprising as these animals usually share the same barn. The observation of clusters with isolates from the same farm but harbouring different ESBL genes may be due to the fact that most of these β-lactamase genes are transported in mobile genetic elements that are prone to rearrange within the genome, which facilitates easy gain or loss of genes.

Most clusters were formed by isolates from different farms (Fig 1). This may be caused by transport of animals or humans, such as farmers or veterinarians that are in close contact with the animals, or these farms may receive young animals from the same source. Unfortunately, no data are available on this. Our results are in contrast to a previous German study in swine farms, were 20 ESBL-*E*. *coli* isolates were analyzed by PFGE and appeared to be grouped by farm [26]. Interestingly, in the latter study isolates were obtained from inside and outside the pig barns indicating that bacteria may have spread into the environment of the stables due to translocation of faeces through pigs, people or vehicles [26].

The most studied and clinically important clone in ESBL-producing *E*. *coli* belongs to sequence type (ST) 131. This highly virulent clone was first described in human clinical isolates, but has also been observed in animal species, such as companion animals, poultry and food [2]. This ST was not found in the present study in the tested isolates, where CC10 (including ST10 and ST167) appeared to be the most prevalent and was associated with *bla*_CTX-M-1, 15; group 9_. CC10 has been previously described in livestock animals [2]. Moreover, ST10 *E*. *coli* isolates have been frequently identified in humans and food products associated with a diversity of ESBL genes, including TEM-52 [15, 19, 50]. In addition, isolates belonging to CC23 (*bla*_CTX-M-15_) and CC38 (*bla*_CTX-M-9 group_) were found in this study. These clonal complexes have also been associated with the production of carbapenemases, being CC38 related to a higher proportion of multiresistant strains [2, 54].

In conclusion, this study adds knowledge about the prevalence of ESBL/AmpC-producing bacteria in German pig holdings including antimicrobial resistance and epidemiological data of a large collection of isolates, although it may have some limitations as the absence of comparison with human isolates and the lack of characterization of plasmids involved. However, it contributes important information governing the implementation of appropriate actions in veterinary medicine to reduce the rate of resistant bacteria among food-producing animals and highlights the importance to perform detailed typing studies of isolates from both humans and animals to prove the possible zoonotic transmission from animal to humans.
